# Calcium and Phosphate Metabolism, Blood Lipids and Intestinal Sterols in Human Intervention Studies Using Different Sources of Phosphate as Supplements—Pooled Results and Literature Search

**DOI:** 10.3390/nu10070936

**Published:** 2018-07-20

**Authors:** Ulrike Trautvetter, Bianka Ditscheid, Gerhard Jahreis, Michael Glei

**Affiliations:** 1 Institute of Nutritional Sciences, Friedrich Schiller University Jena, Dornburger Straße 24, 07743 Jena, Germany; Bianka.Ditscheid@med.uni-jena.de (B.D.); Gerhard.Jahreis@uni-jena.de (G.J.); Michael.Glei@uni-jena.de (M.G.); 2 Institute of General Practice and Family Medicine, Jena University Hospital, Bachstraße 18, 07743 Jena, Germany

**Keywords:** calcium phosphate supplementation, phosphate supplementation, calcium-phosphate complexes, humans, bile acids, blood lipids, mineral metabolism

## Abstract

Phosphates are associated with negative physiological effects. The objectives of this publication were to compare differential effects of supplementation with calcium phosphate or phosphate alone in healthy humans. Four adult human studies were conducted with pentacalcium hydroxy-trisphosphate supplementation (CaP; 90 subjects) and their data were pooled for assessment. For literature search; PubMed and ISI Web of Knowledge were used and 21 items were assigned to three main topics. The pooled study results show that following CaP supplementation, faecal calcium and phosphorus and urinary calcium were increased, blood lipids were positively modulated, and faecal bile acids were increased, as compared with placebo. The literature search reveals that following calcium phosphate supplementation, urinary calcium was increased. Following solely phosphate supplementation, urinary phosphorus was increased and urinary calcium was decreased. Postprandial calcium concentrations were increased following calcium phosphate supplementation. Postprandial phosphate concentrations were increased following solely phosphate supplementation. Calcium phosphate supplementation resulted in rather positively modulated blood lipids and gut-related parameters. The presented results show the relevance to distinguish between calcium phosphate and solely phosphate supplementations, and the importance of a balanced calcium and phosphorus intake.

## 1. Introduction

Phosphorus is one of the most important minerals in human physiology, possessing functions such as structural integrity in bones and teeth, cell signalling, and blood buffering [1,2]; however, both hyperphosphatemia and high intake of phosphorus are discussed very critically in the literature with respect to an increased risk of cardiovascular morbidity and mortality [3,4]. Phosphorus is not only physiologically important, it also fulfils many functions as an additive in food production, like chemical leavening of cakes, structural maintenance and hydration of meat, and flavouring in beverages [5]. Therefore, foods containing phosphate additives range from baked goods to cola beverages, and as such, contribute to the total dietary intake of phosphorus [6,7]. 

Under normal physiological conditions, the absorption of phosphorus by the small intestine is equivalent to that excreted by the kidneys [8]. The physiological postprandial increase in the plasma/serum phosphate concentration is normalised by an increase in renal phosphorus excretion. A number of hormones and factors are involved in phosphorus metabolism, including vitamin D, parathyroid hormone (PTH), and fibroblast growth factor 23 (FGF23). PTH inhibits renal phosphorus reabsorption, resulting in a decrease in the plasma/serum phosphate concentration, while FGF23 regulates high plasma phosphate concentrations by increasing renal phosphorus excretion and decreasing phosphorus absorption in the gut [8].

It is not surprising that phosphorus and calcium metabolism are closely linked, since they share regulatory factors such as Vitamin D (1,25-(OH_2_)D) and PTH. Furthermore, both minerals represent the main components of bone mass, hydroxyapatite; therefore, a chronic imbalance in dietary calcium and phosphorus intake leads to bone loss [9]. In the small intestine, dietary calcium and phosphate are able to form calcium-phosphate complexes (amorphous calcium phosphate, ACP), which can precipitate nutritional ingredients and intestinal substances such as bile acids and fatty acids [10]. Consequently, several physiological changes may occur in the human gut, such as modulation of the composition of faecal bile acids and gut microbiota [11,12,13]. Increased excretion of bile acids can result in an interruption of the enterohepatic circulation. Consequently, the blood cholesterol concentration may decrease due to the de novo syntheses of bile acids from cholesterol in the liver [11,12]. Furthermore, certain bile acids are suspected to be involved in the development of cancer [14]. This potential risk could be reduced by precipitation of bile acids with ACP, since they are no longer able to promote cytotoxic or genotoxic processes [15].

Several studies have shown beneficial modulation of blood lipids, gastrointestinal hormone secretion, gut microbiota, and bile acid excretion by calcium phosphate supplementation [11,12,16,17,18,19]. In these cases, the additional amount of calcium was greater than that of phosphorus. The question therefore arises whether a higher amount of phosphorus leads to similar results. Trautvetter et al. (2018) showed that microbiota and faecal short-chain fatty acids (SCFA) were modulated by phosphate supplementation [13]; however, it was also shown that calcium metabolism was impaired by a high-phosphate diet [20]. Such effects of phosphate supplementation are of particular interest, since the phosphorus intake of Western societies is far above the recommendation, not least due to the usage of phosphate additives in food production [6,21].

The objectives of the present study are to summarise and compare the effects of high phosphorus diets due to calcium phosphate or solely phosphate supplementation regarding metabolism, blood lipids, and intestinal parameters in adults without kidney disease. The presented data combine pooled results of our own randomised human intervention studies with results of randomised human intervention studies from a literature search.

## 2. Materials and Methods 

### 2.1. Study Design and Subjects

**Figure 1:**
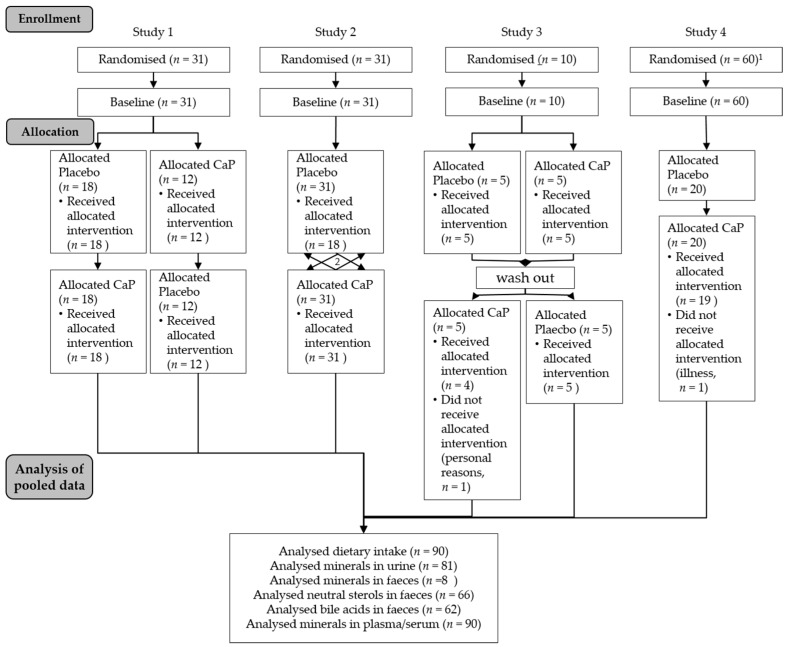
Flow chart of the pooled study course. CaP: Ca_5_(PO_4_)_3_OH pentacalcium hydroxy-trisphosphate; 1. Study 4 was parallel-designed with three arms—for pooled analyses only, the CaP intervention arm was used; 2. Study 2 was cross-over-designed with two further supplements (see Methods section)—for pooled analyses, only the CaP intervention was used.

For the pooled analyses, data of four human intervention studies with pentacalcium hydroxy-trisphosphate (Ca_5_(PO_4_)_3_OH; CaP; cfb Germany;) conducted at our institute were used (hereafter pooled study, Figure 1). A concise overview of the characteristics of the studies with all supplementations and overlapping analyses are shown in Table 1. Results and further information from Studies 1 [11], 3 [19], and 4 [20] are published elsewhere. Results of Study 2 are not published.

All studies were conducted at the Institute of Nutrition of the Friedrich Schiller University Jena. The four studies were performed according to the guidelines laid down in the Declaration of Helsinki; the Ethical Committee of the Friedrich Schiller University Jena (Study 1: 0903-07/02; Study 2: 1828-07/06; Study 3: 2833-05/10; Study 4: 2959-11/10) approved all procedures involving human subjects. Written informed consent was obtained from all subjects. Three trials were registered at ClinicalTrials.gov (Study 2: retrospectively as NCT03286673; Study 3: NCT01296997; Study 4: NCT01297023). Exclusion criteria for all four studies included diseases of the gastrointestinal tract, pregnancy, nursing, and intake of any medication (e.g., for thyroid gland) or dietary supplements. In Study 3, only men took part. The baseline characteristics of the subjects from the pooled study are presented in Table 2.

### 2.2. Supplement

CaP was used as a supplement in all considered studies. In Studies 1, 3, and 4, CaP was incorporated into whole wheat bread to achieve an additional daily calcium and phosphorus intake of 1000 mg and 500 mg, respectively. Participants consumed approximately 135 g of this bread daily. In Study 2, CaP was mixed into whole wheat bread and into Filinchen®, a light and crispy wafer bread. Subjects were required to eat 100 g of the bread and two Filinchen® (approximately 6 g each) daily to achieve the additional calcium intake of 1000 mg. Placebo bread was prepared in exactly the same way, but without the CaP supplement.

### 2.3. Sample Preparation

Sample preparations of Studies 1, 3 and 4 were published elsewhere [11,19,20]. In Study 2, blood samples were drawn by venepuncture and collected in serum. All tubes were centrifuged and the supernatants were stored at −80 °C until analysis. 

Faecal samples of Study 2 were quantitatively collected for 3 days. Each specimen was immediately transported to the study centre, where it was weighed, frozen, and stored at −20 °C. At the end of the study, faecal samples were defrosted, homogenised, portioned, and refrozen (−20 °C) for further storage. The collected 24-h urine (3 days) was transported to the study centre each day. The urine volume from every participant was measured and aliquots were frozen at −20 °C until analysis.

### 2.4. Food Analysis

Food intake of Studies 1, 3 and 4 was analysed as published elsewhere [11,19,20]. In Study 2, subjects documented their normal nutritional habits in a weighed dietary record for 3 days. The intake of macronutrients and minerals from the weighed dietary records were calculated using the Prodi® software (versions 4.5; Nutri-Science GmbH, Freiburg, Germany).

### 2.5. Blood Analysis

Methods of blood analysis of Studies 1, 3 and 4 were published elsewhere [11,19,20]. The analysis of serum minerals in Study 2 was performed using a Synchron LX or CX (Beckman Coulter, Brea, CA, USA), according to the manufacturer’s recommendations.

In Study 2, the analysis of serum total cholesterol, HDL-cholesterol, and triacylglycerides was performed by photometry, following enzymatic preparation, using a SYNCHRON LX or CX. LDL-cholesterol was calculated using the Friedewald formula. 

### 2.6. Faecal and Urine Analysis

Faeces and urine of Studies 1, 3 and 4 were analysed as described previously (except neutral sterols and bile acids) [11,19,20]. In Study 2, mineral concentrations in faeces were determined following pressure digestion using ICP-OES (Optima 3000, Perkin Elmer, Waltham, MA, USA). 

Faecal neutral sterols (cholesterol and its metabolites coprostanol, coprostanone, cholestanol, cholestanone, and cholestenone) and bile acids (iso-lithocholic acid: iLCA, lithocholic acid: LCA, iso-deoxycholic acid: iDCA, deoxycholic acid: DCA, cholic acid: CA, chenodeoxycholic acid: CDCA, and 12keto deoxycholic acid: 12keto DCA) were analysed as described previously [22]. Briefly, neutral sterols were extracted with cyclohexane following a mild alkaline hydrolysis. Analysis was performed by GC-FID (GC17A-AF Vers. 3, Shimadzu, Kyoto, Japan). Bile acids were extracted with diethyl ether following a strong alkaline hydrolysis. Thereafter, extracts were methylated, silylated, and analysed by GC-MS (GC17-QP5000, Shimadzu, Kyoto, Japan).

### 2.7. Absorption and Balance of Calcium and Phosphorus

Absorption (1) and balance (2) of calcium and phosphorus were calculated using the following equations:

(1) $$Absorption\;\left(\%\right)=\frac{Intake\;\left(\frac{mg}{day}\right)-Faecal\;excretion\;\left(\frac{mg}{day}\right)}{Intake\;\left(\frac{mg}{day}\right)}\;\times 100$$

(2) $$Balance\;\left(\%\right)=\frac{Intake\;\left(\frac{mg}{day}\right)-(Urine\;excretion\;\left(\frac{mg}{day}\right)+\;Faecal\;excretion\;\left(\frac{mg}{day}\right)}{Intake\;\left(\frac{mg}{day}\right)}\;\times \;100$$

### 2.8. Statistics

Subjects and samples from each subject were coded to protect volunteer identity and to mask treatment groups during sample collection and analysis. Statistical analysis was performed using the statistical software package IBM SPSS Statistics v24 (SPSS Inc. IBM Company, Chicago, IL, USA). Studies with the same parameters were summarised to increase sample size and reinforce the power of detection of biologically relevant differences. In Study 4, the subjects were supplemented with CaP for 8 weeks. Blood, urine, and faeces were collected after 4 and 8 weeks. To mix similar intervention times for the pooled analyses, the results of Study 4 from the 4-week collection were considered. This is in accordance with the supplementation times of Studies 1, 2 (each 4 weeks), and 3 (3 weeks). The effect of supplementation (placebo vs. CaP) was tested using a paired Students *t*-test. Furthermore, the change between CaP and placebo was tested against zero using a one-way ANOVA and “study” as a random factor. Results are presented as the mean, standard deviation, and 95% confidence interval (CI 95%). Statistical significance is taken at a *p* value of ≤0.05.

### 2.9. Literature Search

**Figure 2:**
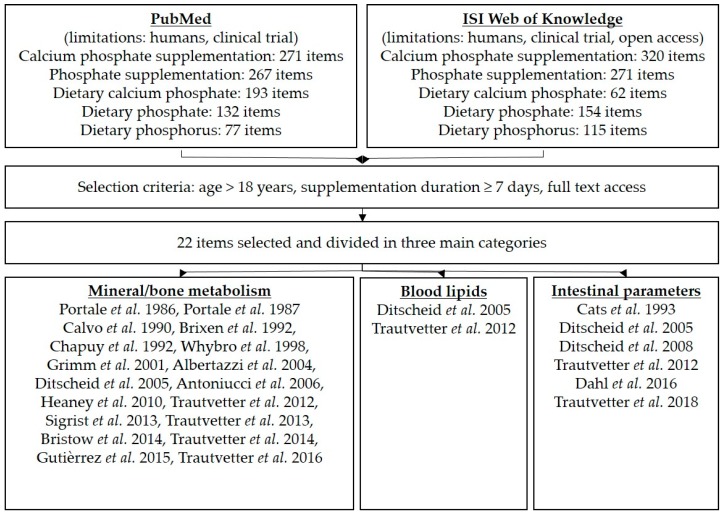
Overview of the literature search and selection criteria.

For the literature search, “PubMed” and “ISI Web of Knowledge” were used with the following search words: “calcium phosphate supplementation” or “phosphate/phosphorus supplementation”. Further criteria were human, clinical trial, age > 18 years, kidney health, duration of supplementation at least seven days, and online availability as a full text. After the final search, all appropriate publications were assigned to three main topics: mineral metabolism, blood lipids, and intestinal parameters (Figure 2). In the results section of the literature search, parameters of the studies are mentioned with respect to phosphorus/phosphate/calcium in plasma/serum, urine, or faeces; blood lipids; bile acids/neutral sterols in faeces; and further intestinal parameters (SCFA, microbiota). In certain publications using tricalcium phosphate supplementation, phosphorus intake was not stated; therefore, we estimated the phosphorus intake based on the molecular formula for tricalcium phosphate: Ca_3_(PO_4_)_2_.

## 3. Results

### 3.1. Results of the Pooled Study

#### 3.1.1. Dietary Intake of Macronutrients and Minerals

Dietary intake of energy, fat, protein, and carbohydrates did not change as a result of CaP supplementation compared with placebo (Table 3); however, calcium and phosphorus intake were significantly increased following CaP supplementation (Table 3).

#### 3.1.2. Minerals

Following placebo and CaP supplementation, the plasma/serum concentrations of calcium (placebo: 2.40 ± 0.14 mmol/L, CI95%: 2.37–2.43 mmol/L; CaP: 2.39 ± 0.15 mmol/L; CI95%: 2.36–2.42 mmol/L) and phosphate (placebo: 1.27 ± 0.19 mmol/L, CI95%: 1.23−1.31 mmol/L; CaP: 1.29 ± 0.20 mmol/L; CI95%: 1.25–1.33 mmol/L) were not significantly different (data not shown). 

The absorption and balance of calcium (absorption: placebo: 21 ± 32%, CI95%: 14–28%; CaP: 23 ± 16%; CI 95%: 18–29%; balance: placebo: 7 ± 32%, CI 95%: 0–14%; CaP: 15 ± 26%; CI 95%: 9–21%) were not significantly different after CaP supplementation compared to placebo (data not shown). Phosphorus absorption was significantly lower (placebo: 58 ± 18%, CI95%: 54−62%; CaP: 52 ± 18%; CI 95%: 48–56%) and phosphorus balance (placebo: 3 ± 29%, CI 95%: −3–10%; CaP: 10 ± 23%; CI 95%: 5–15%) was significantly higher after CaP supplementation compared to placebo (data not shown). 

**Figure 3:**
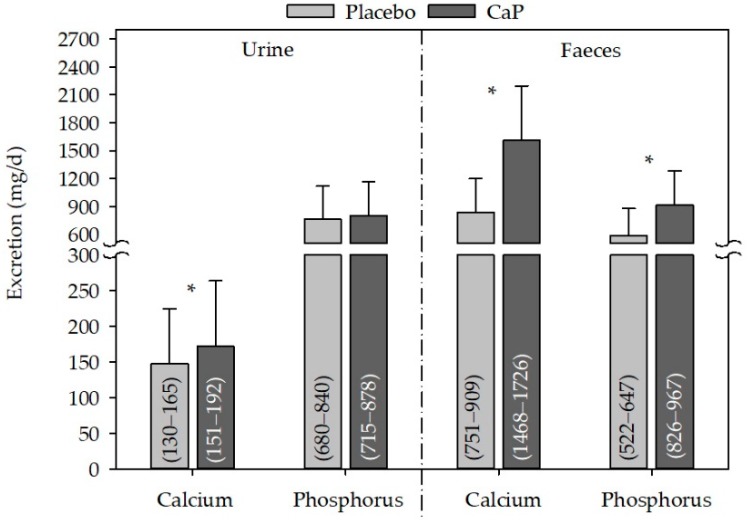
Effect of CaP supplementation on urine and faecal calcium and phosphorus excretion after CaP supplementation. *n* (studies) = 3; *n* (subjects) = 81; mean + standard deviation; (95% confidence interval); * significantly different to placebo (paired Student’s *t*-test, *p* ≤ 0.05); CaP: three–four weeks’ intervention with Ca_5_(PO_4_)_3_OH (pentacalcium hydroxy-trisphosphate).

Urinary calcium excretion was significantly increased following CaP supplementation as compared with placebo (Figure 3), and the change between CaP and placebo was also significant (24 ± 65 mg/day, CI95%: 10−39 mg/day, *p* ≤ 0.05). Phosphorus excretion in the urine was not modified by supplementation with CaP as compared with placebo. In contrast, in faeces, both calcium and phosphorus excretion were increased following CaP supplementation as compared with placebo (Figure 3), and the change between CaP and placebo was also significant (calcium: 773 ± 580 mg/day, CI95%: 646–896 mg/day, *p* ≤ 0.05; phosphorus: 325 ± 344 mg/day, CI95%: 251−399 mg/day, *p* ≤ 0.05).

#### 3.1.3. Blood Lipids

Total cholesterol and LDL-cholesterol concentration as well as the LDL:HDL ratio, were significantly decreased following CaP supplementation as compared with placebo. Considering the changes, only the change in LDL concentration was significant (Table 4). Plasma/serum concentrations of HDL-cholesterol and triacylglycerides showed no modifications following CaP supplementation as compared with placebo (Table 4).

#### 3.1.4. Faecal Sterols

**Figure 4:**
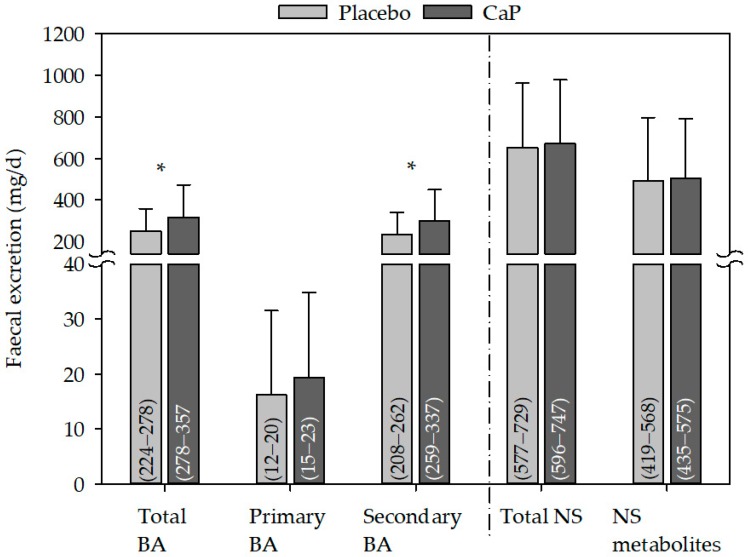
Effect of CaP supplementation on faecal excretion of bile acid and neutral sterols. BA: *n* (studies) = 2; *n* (subjects) = 62; NS: *n* (studies) = 3; *n* (subjects) = 66; mean + standard deviation; (95% confidence interval); * significantly different to placebo (paired Student’s *t*-test, *p* ≤ 0.05); CaP: three–four weeks’ intervention with Ca_5_(PO_4_)_3_OH (pentacalcium hydroxy-trisphosphate); BA: bile acids; NS: neutral sterols.

The excretion of total and secondary bile acids in faeces were both significantly increased following CaP supplementation as compared with placebo (Figure 4), and the change between CaP and placebo was also significant (total bile acids 67 ± 120 mg/day; CI 95%: 36–97, *p* ≤ 0.05; secondary bile acids: 63 ± 116 mg/day, CI 95%: 34−93, *p* ≤ 0.05). This was likely due to the significant increase in LCA, DCA, and 12keto DCA following CaP supplementation as compared with placebo (Table 5). 

The excretion of neutral sterols in faeces did not change following CaP supplementation (Figure 4, Table 6).

Gender-specific analysis revealed mainly similar results, which are presented in Tables S1−S4 and Figures S1−S4.

### 3.2. Results of the Literature Search

The literature search yielded 940 results in the PubMed and 922 in the ISI Web of Knowledge database (Figure 2). After selection with the above mentioned criteria, 22 items were used and assigned to the three main topics: mineral metabolism (18 items), blood lipids (two items) and intestinal parameters (six items). Study characteristics and results of the used literature items are presented in Table 7.

#### 3.2.1. Metabolism of Phosphorus and Calcium

Serum phosphate concentrations were not modified in two studies [18,19] and increased [25] or decreased in one study [26], respectively, after calcium phosphate supplementations. One study showed an increase [25] and one study showed no change [19] in urinary phosphorus after supplementation with calcium phosphate. A decrease of urinary phosphorus was not shown in the selected studies. In three studies, faecal phosphorus excretions increased after calcium phosphate supplementation [11,12,19]. Serum calcium concentration increased in two studies (increase in the postprandial area under the curve by [18] and increase in fasting concentration by [19]) and did not changed in four studies after calcium phosphate supplementation [11,18,24,25]. Calcium phosphate supplementation led to increased urinary calcium in four studies [11,19,23,25]. None of the selected studies showed no change or a decrease in urinary calcium. Faecal calcium excretion was increased in three studies due to calcium phosphate supplementation [11,12,19]. 

The supplementation with phosphate alone led to an increase of serum/plasma phosphate concentration in one study ([30]: 24 h mean) and to a decrease in serum/plasma phosphate concentration in one another study [29]; whereas eight studies did not show an effect on serum/plasma phosphate concentration [20,31,32,33,34,35,36,37]. Urinary phosphorus excretion showed an increase in six studies [20,29,32,33,34,35] and a decrease in one study [36] after solely phosphate supplementation. Faecal phosphorus excretion was increased in one study after solely phosphate supplementation [20]. Serum/plasma calcium concentration remained unchanged after solely phosphate supplementation in eight studies [20,29,30,31,32,33,34,36]; no study showed an increase or a decrease. Urinary calcium showed a decrease in three studies [20,29,33] and four studies revealed no effect [32,34,35,36] after solely phosphate supplementations. Regarding faecal calcium concentrations, one study reported no changes after phosphate supplementation [20].

#### 3.2.2. Blood Lipids

Only two studies determined the effect of calcium phosphates on blood lipid concentrations. Both studies showed a decreased cholesterol concentration [11,12] and one study showed a decreased LDL-cholesterol concentration [12]. 

#### 3.2.3. Intestinal Parameters

Two studies showed increased faecal excretions of bile acids after calcium phosphate supplementation [11,12]. In faecal water, one study showed decreased concentrations of iLCA, total neutral sterols, cholesterol and its metabolites and no modulations of genotoxicity after calcium phosphate supplementation [16]. Increased faecal lactobacilli and supplemented probiotic strains were found in two studies after calcium phosphate supplementation [12,27]. One study showed unaffected intestinal parameters like cytolytic activity of fecal water or bile acids in faeces after calcium phosphate supplementations [28].

The genotoxicity and cytotoxicity of faecal water remained unchanged after supplementation with phosphate alone in one study [13].

## 4. Discussion

The beneficial modulation of the intestinal environment by calcium phosphate supplementation has been shown several times [11,12,27]. These effects may be attributed to the formation of calcium-phosphate complexes and the resulting precipitation and increased excretion of intestinal substances. The formation of calcium complexes in the human gut was first mentioned in the early 1980s, and was supposed to be a complex of calcium ions, bile acids and fatty acids [38]. Some years later, Van der Meer et al. (1990) showed that phosphate was not a competitor in the precipitation of calcium with bile acids, but rather a necessity for the formation of ACP [39]. The complexation occurs in the small intestine, at a pH value of 5.6 and a molar calcium-to-phosphate ratio of 3:2 [40]. The complexed calcium and phosphate ions are not absorbed, and thus, are unavailable to the body. The following subsections compare and summarise the effects of calcium phosphate with those of phosphate supplementation alone, with respect to their metabolism and the effects on blood lipids and intestinal parameters.

### 4.1. Metabolism of Calcium and Phosphorus

The results of our pooled study reveal significantly increased faecal calcium and phosphorus excretion following supplementation with CaP as compared with placebo. Trautvetter et al. (2016) reported significantly increased faecal phosphorus concentrations after solely phosphate supplementation, as well as significantly increased faecal calcium and phosphorus concentrations after combinatory supplementation with phosphate and calcium carbonate [20]. Other studies found in the literature search for calcium phosphate and solely phosphate supplementation did not determine faecal excretion of calcium and phosphorus. Based on the studies conducted in our institute, it appears that the main portion of the supplemented calcium and phosphate is excreted via the faeces, maybe as part of an ACP complex.

The main excretion organs for calcium and phosphorus are the kidneys. Changes in the absorption rates of calcium and phosphorus should result in modulated urinary excretion. Our pooled study results show a significantly increased urinary calcium excretion, but an unchanged urinary phosphorus excretion following CaP supplementation as compared with placebo. In contrast, Albertazzi et al. (2004) and Heaney et al. (2010) reported significant increases in urinary phosphorus concentration following ossein-hydroxyapatit/tricalcium phosphate and tricalcium phosphate supplementation, respectively [23,25]. Heaney et al. (2010) stated that most absorbed phosphorus would be spilled into the urine and concluded that the relatively small increase in urine due to tricalcium phosphate supplementation suggests that the absorbed phosphorus concentration was low. This was likely caused by the complexing of phosphorus with calcium in the salt [25]. 

In almost all studies that measured urinary phosphorus, phosphate supplementation alone led to significantly increased urinary phosphorus excretion [20,29,32,33,34,35], with the exception of a study by Guitérrez et al. (2015), in which urinary phosphorus was significantly decreased following a one-week high-phosphorus additive diet as compared with baseline [36]. However, it must be noted, that in this study the high-phosphorus diet followed a one-week low-phosphorus additive diet, and it is possible that this had an effect on the results of the high-phosphorus diet. Regarding urinary excretion, the studies by Portale et al. (1986), Whybro et al. (1999), and Trautvetter et al. (2016) showed significant decreases of calcium following phosphate supplementations [20,29,33]. This effect could be a hint of a diminished calcium metabolism, especially when calcium intake remained constant (approximately: 1000 mg calcium/day) [20,33,41]. Zemel et al. (1981) showed that urinary calcium excretion decreased due to phosphate supplementations (at low and high calcium intakes). The decrease was lower, when calcium intake was high. Furthermore, they demonstrated that calcium balance was negative after low calcium and high orthophosphate supplementation; calcium equilibrium was achieved only after high calcium and high orthophosphate diets. They also showed, that urinary calcium decreased after high phosphate diets due to increased fractional renal tubular reabsorption of calcium and not because of decreased calcium absorption [42]. The increase in fractional renal tubular reabsorption of calcium was also shown in a study with high and low protein intakes and additional high and low phosphate intakes. The increase in phosphate intake caused a decrease in urinary calcium, an increase in fractional renal tubular reabsorption of calcium at high and low protein intakes, but a negative calcium balance when protein intake was high and phosphate intake was low [43]. 

The above presented results allow the following assumptions. Firstly, if a calcium phosphate compound is supplemented, urinary calcium will increase without an increase in phosphorus excretion; which may be the result of partly absorbed calcium, but not phosphate. This effect is very small, since calcium absorption and balance did not change. The increased faecal excretion of calcium and phosphate may be caused by ACP formation. Secondly, if phosphate is supplemented alone, it will be well absorbed, it increases urinary phosphorus excretion and decreases calcium excretion partially. 

Fasting calcium and phosphate concentrations were not influenced by supplementation in most of the studies or in our pooled analyses (unchanged calcium and phosphate concentrations: [26,29,31,32,33,34,35,37]). These results are independent of the duration of dosing (7 till 10 days of supplementation [29,32,33,34,35] and 6 weeks till 18 months of supplementation [24,26,31,37]). This reflects the tight regulation of calcium and phosphate homeostasis in the blood. The postprandial measurements in one study demonstrated that the area under the curve of calcium and phosphate concentrations following 3 weeks of CaP supplementation was increased or remained unchanged, respectively, as compared with placebo; therefore, CaP may contribute to an adequate calcium supply [18]. Chapuy et al. (1992) highlighted that the favourable calcium:phosphorus ratio of the tricalcium phosphate supplement and phosphate supplementation itself have positive effects on calcium absorption [24]. This is in accordance with the above-mentioned assumption that calcium, but not phosphate, of the calcium phosphate compound is more absorbed in the small intestine.

In contrast, supplementation with a phosphate compound mixture by Portale et al. (1987) showed a significant increase in the mean 24-h serum phosphate concentration, but a decreased mean 24-h serum ionized calcium concentration (total calcium unchanged) [30]. These results strengthen the above-mentioned assumption that phosphate supplementation alone or a high phosphate intake, respectively, maybe diminishes calcium metabolism, especially when calcium intake is low or remains constant [41,42,44]. 

The question arises, therefore, whether the modulated absorption of calcium by calcium phosphate and phosphate supplementation leads to changes in bone health. Most of the studies revealed positive effects on markers of bone health following calcium phosphate supplementation [19,23,24,25,26]. Following phosphate supplementation alone, certain studies demonstrated no modulation of determined markers [20,32,33,37]; however, Gutiérrez et al. (2015) demonstrated a disturbed bone metabolism due to increasing osteocalcin and osteopontin concentrations, as well as decreasing sclerostin concentrations, following high-phosphate diets [36]. Despite the fact that not all phosphate supplementation studies have revealed negative effects on bone metabolism, the presented results show the importance of a balanced intake of phosphorous and calcium.

### 4.2. Bile Acids and Blood Lipids

Positively charged calcium ions are located on the surface of ACP. Through ionic absorption between them and the negatively charged carboxyl group of glycine-conjugated and free bile acids, precipitates will be formed. Thus, these bile acids are unavailable to enterohepatic circulation Furthermore, the exposure of the hydrophobic tail of the bile acids to the aqueous phase of the faecal bulk leads to the formation of further hydrophobic aggregates, which are able to bind other hydrophobic ligands, for instance, neutral sterols [45]. Our pooled study results show a significant increase in faecal total and secondary bile acid excretion following CaP supplementation as compared with placebo. Apart from our studies [11,12,16], only a study by Cats et al. (1993) investigated the effect of calcium phosphate supplementation on faecal bile acid composition; however, no effects were observed [28]. It can be assumed that ACP and bile acids form hydrophobic aggregates, which was originally proposed by Govers et al. (1994) [16,46]. This precipitation leads to increased bile acid concentrations in faeces and decreases those in faecal water [16]. Furthermore, the modulation of faecal neutral sterols (faeces and faecal water) has been proposed to be a direct co-precipitation at the surface of the ACP-bile acid complex or a direct interaction between neutral sterols and ACP [16]. The bile acid binding ability to ACP in the small intestine has also been shown in both in vitro and in vivo studies with calcium carbonate or milk calcium supplementation [10,39,47,48]. 

Bile acid precipitation and the resulting disruption of enterohepatic circulation may result in the lowering of blood lipid concentrations. The results of our pooled study reveal a significant decrease in the total and LDL-cholesterol concentrations in plasma/serum and a significant reduction in the LDL:HDL ratio following CaP supplementation as compared with placebo. There are no further available studies that focused on blood lipid levels following calcium phosphate or phosphate supplementation. Reid et al. (2004) dealt with calcium supplementation on blood lipid levels and postulated possible mechanisms of action [49]. Firstly, calcium directly interacts with fatty and bile acids, and secondly, supplemented calcium has an effect on calcitropic hormones. This could lead to a decrease in 1,25-(OH)_2_D and PTH concentrations, and thus, result in the promotion of lipolysis in adipocytes [49]. In two other studies evaluating calcium phosphate supplementation, the PTH concentrations were decreased [24,26]. A high-phosphate diet, however, is associated with increased PTH concentrations [44]. 1,25-(OH)_2_D was decreased following CaP supplementation, but increased following phosphate supplementation as compared with the respective placebo [19,20]. Therefore, a blood lipid-reducing effect by calcitropic hormones following calcium phosphate supplementation is conceivable, but implausible following phosphate supplementation.

### 4.3. Toxicological Aspects

Consumption of calcium phosphate or phosphate supplements increases the mineral amounts of phosphorus and calcium in the human gut. Therefore, it is worth considering potential adverse effects. Weiner et al. (2001) referred to the consequences of chronic high-phosphate loads in rats, dogs and rabbits following the feeding of inorganic phosphates for 21–104 weeks [50]. High intakes of inorganic phosphates led to kidney damage, bone demineralisation, and the release of calcium from bones. Furthermore, excessive phosphate and calcium loads resulted in nephrocalcinosis and other renal effects. The authors determined a no observed effect level (NOEL)/no observed adverse effect level (NOAEL) of inorganic phosphates of 225 mg/kg/day. Assigned to humans, an acceptable daily intake (ADI) was established of 63 mg phosphorus/kg/day [50]. This ADI calculation took into account that rats are more susceptible to renal calcification caused by inorganic phosphates [50]. The data of our pooled study show a mean phosphorus intake of 28 ± 5 mg/kg body weight following CaP supplementation and 21 ± 5 mg/kg body weight following placebo. These values are far below the ADI, but with daily intakes of 1434 ± 315 mg (placebo) and 1924 ± 323 mg (CaP), above the recommended daily intake of 700 mg/day [51]. 

Our group focused on the effects of greater amounts of phosphorus and calcium in the gut on the genotoxicity and cytotoxicity of faecal water. In a study by Ditscheid et al. (2009), CaP supplementation did not lead to a faecal water-induced increase in DNA strand breaks, as measured by the alkaline comet assay [16]. Cats et al. (1993) also showed that there was no change in the cytolytic activity of faecal water following calcium phosphate supplementation [28]. Trautvetter et al. (2018) showed that there were no changes in the genotoxic potential or the cytotoxicity of faecal water following phosphate supplementation [13]. All these authors supposed that the formation of calcium phosphate complexes and the co-precipitation of bile acids or other intestinal substances may be responsible for a rather decreased toxicity. However, beneficial effects regarding the genotoxicity and cytotoxicity of faecal water were not shown.

Interestingly, Dahl et al. (2012) and Trautvetter et al. (2012) reported modulations of the microbiota following combined supplementation of calcium phosphate with a probiotic strain [12,27]. The authors concluded that calcium phosphate co-administration may increase gastrointestinal survival of the probiotic strains, and that this may be caused by a shift towards a less harmful intestinal environment due to the formation of ACP [12,27]. These two human studies confirmed previous results obtained in animals. Bovee-Oudenhoven et al. (1999) showed that calcium phosphate stimulates the growth of lactobacilli and improves the prevention of salmonella infections [52]. Thus, it can be concluded that supplementation with calcium phosphate does not modify the postulated genotoxic effects of faecal water, but likely modulates the intestinal environment by ACP formation.

### 4.4. Limitations

By interpreting the data, it should be emphasized that subjects of the presented results from the pooled human study as well as from the literature search are subjects free of kidney diseases. Kidney diseases are characterized by an impaired glomerular filtration rate and in the first stages of chronic kidney disease (CKD) the phosphate concentration in plasma is maintained by PTH and FGF23. But, when glomerular filtration rate continues to fall (<50 mL/min), compensatory mechanisms fail and this leads to hyperphosphatemia [53,54]. Hyperphosphatemia is strongly associated with increased cardiovascular morbidity and mortality in patients with CKD [55]. It is known that phosphate homeostasis is impaired very early in the course of CKD and that patients with moderate CKD (but with normal phosphate concentrations in plasma) have an increased mortality risk [56]. Presently, CKD is underdiagnosed and undertreated [57]

Therefore, it is possible that the effects of calcium phosphate and solely phosphate supplementations are different when looking at adults in the first stages of CKD. 

Limitations of our pooled analysis are the combination of studies with defined diets and individual diets during sample collection and the mix up of plasma and serum. 

Limitations of the studies obtained from the literature search are that there was no parallel design of certain studies, especially older ones, which could lead to carry-over effects. Furthermore, sample sizes were often small, and no power calculations and no molecular formulas for the supplements were presented.

Limitation at review-level is the inclusion of publications with full-text availability only.

## 5. Conclusions

All in all, the present results clearly show that it is relevant to distinguish between calcium phosphate and solely phosphate supplementation. Calcium phosphate supplementation contributes to the calcium supply without an adverse increase in phosphorus absorption. Furthermore, calcium phosphate supplementation leads to increased bile acid excretion, decreased blood lipids, and modulation of the intestinal environment, likely through ACP formation in the small intestine. In contrast, solely phosphate supplementation is associated with an increased absorption of phosphorus and there are hints for a potential diminishing effect on calcium metabolism, especially when calcium intake is low. This might be a problem with regard to the high phosphorus intake as a result of the high consumption of phosphate-rich foods in Western societies, especially when the calcium intake is below the recommendations. Therefore, a balanced calcium and phosphorus intake is recommended. Further research is needed regarding phosphate intakes and effects in first stage CKD patients and health-related consequences of long-term high phosphate intakes. 

## Figures and Tables

**Table 1:** Overview of the characteristics of the four studies used in the pooled analyses (used interventions highlighted in grey).

	Study 1	Study 2	Study 3	Study 4
**Study characteristics**				
Design	Double-blind, placebo-controlled			
	Cross-over	Cross-over	Cross-over	Parallel
Year	2002	2006	2010	2011
Supplement, dosage and duration	Ca_5_(PO_4_)_3_OH, 0.5 g P/day 1.0 g Ca/day, 4 weeks	Ca_5_(PO_4_)_3_OH, 0.5 g P/day 1.0 g Ca/day, 4 weeks	Ca_5_(PO_4_)_3_OH, 0.5 g P/day 1.0 g Ca/day, 3 weeks	Ca_5_(PO_4_)_3_OH, 0.5 g P/day 1.0 g Ca/day, 8 weeks
		CaCO_3_, 1.0 g Ca/day, 4 weeks		Vitamin D_3_, 10 µg/day, 8 weeks
		Ca_3_(PO_4_)_2_, 0.52 g P/day 1.0 g Ca/day, 4 weeks		Ca_5_(PO_4_)_3_OH + Vitamin D_3_ 0.5 g P/day 1.0 g Ca/day, 10 µg/day vitamin D3, 8 weeks *
	Placebo, 4 weeks	Placebo, 4 weeks	Placebo, 3 weeks	Placebo, 2 weeks
**Analyses**				
Minerals in serum/plasma	✓	✓	✓	✓
Minerals in urine/faeces	✓	✓		✓
Blood lipids	✓	✓		✓
Faecal sterols	✓	✓		✓**

Ca_5_(PO_4_)_3_OH: pentacalcium hydroxy-trisphosphate; Ca_3_(PO_4_)_2_: beta-tricalcium phosphate; CaCO_3_: calcium carbonate; Ca: calcium, P: phosphorus, grey fields indicate the interventions used for pooled analysis, * to mix similar intervention times, the results from four-week collection were considered for the pooled analyses, ** only neutral sterols.

**Table 2:** Baseline characteristics of the pooled study population.

Characteristics	All	Women	Men
*n*	90	42	48
Age (year)	28 ± 9	29 ± 10	27 ± 8
BMI (kg/m^2^)	23 ± 3	22 ± 4	23 ± 3

*n* (studies) = 4, supplement: pentacalcium hydroxy-trisphosphate (CaP) against placebo, duration of intervention: three-four weeks.

**Table 3:** Dietary intake of macronutrients as well as calcium and phosphorus after CaP supplementation.

Parameter	Placebo	CaP	Change
Energy	9 ± 2	9 ± 2	013 ± 1
(MJ/day)	(7–9)	(9–10)	(−0.09–0.3)
Fat	84 ± 25	85 ± 27	0.7 ± 15
(g/day)	(79–90)	(79–91)	(−2–4)
Protein	82 ± 19	82 ± 19	0.3 ± 12
(g/day)	(78–86)	(78–86)	(−2–3)
Carbohydrates	265 ± 49	271 ± 56	5 ± 36
(g/day)	(255–276)	(259–282)	(−3–13)
Calcium	1069 ± 289	2088 * ± 314	1019 # ± 209
(mg/day)	(1009–1130)	(2022–2154)	(975–1063)
Calcium	16 ± 4	31 * ± 6	15 # ±4
(mg/kg bw.)	(15–17)	(30–32)	(14–16)
Phosphorus	1434 ± 315	1924 * ± 323	490 # ± 199
(mg/day)	(1368–1500)	(1857–1992)	(449–532)
Phosphorus	21 ± 4	28 * ± 5	7 # ± 3
(mg/kg bw.)	(20–22)	(27–29)	(7–8)

*n* (studies) = 4; *n* (subjects) = 90; mean ± standard deviation; (95% confidence interval); CaP: three–four weeks’ intervention with Ca_5_(PO_4_)_3_OH (pentacalcium hydroxy-trisphosphate); bw body weight; * significantly different to placebo (paired Student’s *t*-test, *p* ≤ 0.05); # significantly different to zero (one-way ANOVA, *p* ≤ 0.05).

**Table 4:** Effects of CaP supplementation on blood lipids.

Parameter	Placebo	CaP	Change
Total cholesterol	4.80 ± 1.06	4.65 * ± 1.03	−0.15 ± 0.47
(mmol/L)	(4.6–5.0)	(4.4–4.9)	(−0.25–(−0.04))
HDL-cholesterol	1.45 ± 0.37	1.48 ± 0.38	0.03 ± 0.17
(mmol/L)	(1.4–1.5)	(1.4–1.6)	(−0.01–0.07)
LDL-cholesterol	2.87 ± 0.94	2.76 * ± 0.92	−0.11 # ± 0.42
(mmol/L)	(2.7–3.1)	(2.6–3.0)	(−0.2–(−0.02))
LDL:HDL-ratio	2.12 ± 0.91	1.99 * ± 0.84	−0.13 ± 0.41
	(1.9–2.3)	(1.8–2.2)	(−0.2–(−0.04))
Triacylglycerides	0.99 ± 0.59	0.96 ± 0.37	−0.03 ± 0.54
(mmol/L)	(0.9–1.1)	(0.9–1.0)	(−0.2–0.1)

*n* (studies) = 3; *n* (subjects) = 81; mean ± standard deviation; (95% confidence interval); CaP three–four weeks’ intervention with Ca_5_(PO_4_)_3_OH (pentacalcium hydroxy-trisphosphate); * significantly different to placebo (paired Student’s *t*-test, *p* ≤ 0.05); ^#^ significantly different to zero (one-way ANOVA, *p* ≤ 0.05).

**Table 5:** Effect of CaP supplementation on faecal excretion of bile acids.

Parameter	Placebo	CaP	Change
iLCA	39 ± 24	45 * ± 28	6 ± 21
(mg/day)	(33–45)	(38–52)	(0.3–11.0)
LCA	54 ± 31	69 * ± 36	15 # ± 24
(mg/day)	(47–62)	(60–78)	(8.4–20.8)
iDCA	34 ± 24	37 ± 40	3 ± 28
(mg/day)	(28–40)	(27–47)	(−4.1–9.9)
DCA	98 ± 43	135 * ± 73	36 ± 56
(mg/day)	(88–109)	(116–153)	(22.2–50.5)
CDCA	6 ± 4	7 * ± 5	1 # ± 4
(mg/day)	(5–7)	(6–8)	(0.02–2.3)
CA	10 ± 12	12 ± 11	2 ± 11
(mg/day)	(7–13)	(9–15)	(−0.9–4.8)
12keto DCA	8 ± 7	11 * ± 12	4 ± 9
(mg/day)	(6–9)	(8–15)	(1.5–6.0)

*n* (studies) = 2; *n* (subjects) = 62; mean ± standard deviation; (95% confidence interval); * significantly different to placebo (paired Student’s *t*-test, *p* ≤ 0.05); # significantly different to zero (one-way ANOVA, *p* ≤ 0.05); CaP: three–four weeks´ intervention with Ca_5_(PO_4_)_3_OH(pentacalcium hydroxy-trisphosphate); BA: bile acids, iLCA: iso-lithocholic acid; LCA: lithocholic acid; iDCA: iso-deoxycholic acid; DCA: deoxycholic acid; CDCA: chenodeoxycholic acid, CA: cholic acid, 12keto DCA: 12keto-deoxycholic acid; primary BA: sum of CDCA and CA; secondary BA: sum of iLCA, LCA, iDCA, DCA and 12keto DCA.

**Table 6:** Effect of CaP supplementation on faecal excretion of neutral sterols.

Parameter	Placebo	CaP	Change
Coprostanol	430 ± 265	443 ± 259	160 ± 152
(mg/day)	(365–496)	(379–506)	(−47–71)
Cholesterol	160 ± 153	166 ±187	6 ± 121
(mg/day)	(123–198)	(120–212)	(−24–36)
Cholestanol	14 ± 8	13 ± 9	−0.5 ± 5
(mg/day)	(12–16)	(11–16)	(−1.6–0.7)
Coprostanone	41 ± 43	40 ± 33	−1.0 ± 34
(mg/day)	(30–51)	(32–48)	(−9.3–7.3)
Cholestanone	3 ± 3	3 ± 2	0.1 ± 2
(mg/day)	(2–4)	(3–4)	(−0.4–0.6)
Cholestenone	5 ± 4	5 ± 4	0 ± 3
(mg/day)	(4–6)	(4–6)	(−0.8–0.9)

*n* (studies) = 3; *n* (subjects) = 66; mean ± standard deviation; (95% confidence interval); CaP: three–four weeks´ intervention with Ca_5_(PO_4_)_3_OH (pentacalcium hydroxy-trisphosphate).

**Table 7:** Study characteristics and results found in the literature search.

	Study Design	Results ^1^	Comments/More Results ^2^
**Calcium Phosphate Supplementation**			
[23] A	-153 postmenopausal women, >60 years -double-blind, placebo-controlled, parallel design -group TCP: ossein-hydroxyyapatit (TCP; aCa: 500 mg, bCa: 651 mg, aP: 258 mg *) -group OHC: tricalcium phosphate (OHC, aCa: 500 mg, bCa: 563 mg, aP: 258 mg *) -group placebo (bCa: 679 mg) -6 months (analysis after 3 and 6 months)	-uCa↑ after both supplements, OHC > TCP	-TCP is the ash of OHC, OHC included inorganic matrix -markers of bone formation ↓ after TCP and OHC -bone resorption marker and bone mineral density = after TCP and OHC
[24] A	-3270 healthy older women, 84 ± 6 years -placebo-controlled, parallel design -group tricalcium phosphate (aCa: 1,2 g, bCa: 511 mg + 20 µg, aP: 620 mg *, vitamin D_3_) -group placebo: (bCa: 514 mg) -18 months	-sCa = in tricalcium phosphate group -sCa ↓ in placebo group	-hip fractures and nonvertebral fractures ↓ in tricalcium phosphate group -PTH ↓ in tricalcium phosphate group -1,25-(OH)_2_D ↑ in the tricalcium phosphate group -bone density of proximal femur ↑ in tricalcium phosphate group and ↓ in placebo group
[11], A, B, C	-double-blind, placebo-controlled, cross-over design -31 healthy young women and men, 25 ± 2 years -test diet: CaP (aCa: 1000 mg; bCa: 1104 mg, aP: 500 mg, bP: 1498 mg) -placebo diet (bCa: 1193 mg, bP: 1528 mg) -4 weeks each diet	-sCa = after CaP and placebo -uCa, fCa, and fP ↑ after CaP -total cholesterol ↓ after CaP -fBA (total and secondary) ↑ after CaP -faecal cholestenone and cholesterol ↑ after CaP -faecal coprostanol and cholestanol ↓ after CaP	
[16] A, C	-31 healthy young women and men, 25 ± 2 years -double-blind, placebo-controlled, cross-over design -test diet: CaP (aCa: 1000 mg; bCa: 1104 mg, aP: 500 mg, bP: 1498 mg) -placebo diet (bCa: 1193 mg, bP: 1528 mg) -4 weeks each diet	-faecal LCA; DCA and 12keto DCA ↑ after CaP -iLCA in faecal water ↓ after CaP -total NS ↓ after CaP -genotoxicity of faecal water = after CaP	
[25] A	-211 postmenopausal osteoporotic women, 60–85 years -randomised, positive-comparator, 2-arm, single-blind -arm 1: tricalcium phosphate salt (aCa: 1800 mg, aP: 930 mg *, bP: <1000 mg) + 20 µg/day teripatide (osteoporosis therapy) + 1000 IU vitamin D -arm 2: calcium carbonate (aCa: 1800 mg, bP: < 1000 mg) + 20 µg/day teripatide (osteoporosis therapy) + 1000 IU vitamin D -12 months	-sPO_4_ ↑ after tricalcium phosphate supplementation (also calcium carbonate group) -uP/Crea and uCa/Crea ↑ after tricalcium phosphate supplementation -sCa=	-bone resorption marker ↑ in both groups
[12] A, B, C	-32 men and women, 25 ± 5 years -double-blind, placebo-controlled, cross-over design -test diet 1: CaP + probiotic (10^10^ CFU/day) (aCa: 1000 mg; bCa: 807 mg, aP: 500 mg, bP: 1316 mg) -test diet 2: probiotic (10^10^ CFU/d) (bCa: 873 mg, bP: 1379 mg) -placebo diet (bCa: 866 mg, bP: 1367 mg) -4 weeks each diet	-fCa and fP ↑ after CaP + probiotic -total cholesterol and LDL-cholesterol ↓ after CaP + probiotic -f secondary BA ↑ after CaP + probiotic -faecal lactobacilli and supplemented probiotic strain ↑ after CaP + probiotic and probiotic	
[18] A	-9 young men, 27 ± 4 years -double-blind, placebo-controlled, cross-over design -test diet: CaP (aCa: 1000 mg, bCa: 915, aP: 500 mg, bP: 1531 mg) -placebo diet (bCa: 926 mg, bP: 1542 mg) -3 weeks each diet	-sPO_4_ und sCa= after CaP -AUC pCa after three weeks CaP ↑ -AUC pPO_4_ after three weeks CaP=	
[26], A	-100 postmenopausal women, 71 ± 5 years -single-blind, placebo-controlled, parallel design -group 1: calcium citrate (aCa: 1000 mg, bCa: 970 mg) -group 2: calcium carbonate (aCa: 1000 mg, bCa: 810 mg) -group 3: microcrystalline hydroxyapatite preparation 1 (MCHA, aCa: 1000 mg, bCa: 890 mg) -group 4: microcrystalline hydroxyapatite preparation 2 (MCHB, aCa: 1000 mg, bCa: 780 mg) -placebo group (bCa: 900 mg) -3 months	-sPO_4_ after MCHA/MCHB supplementation ↓ as compared with baseline	-no differences after 3 months between calcium carbonate and citrate or between the two MCH preparations → citrate and carbonate as well as 2 MCH preparations were pooled and analysed together -sCa ↑ after citrate/carbonate supplementation -PTH ↓ after all interventions as compared with placebo -bone markers ↓ after all interventions as compared with placebo
[19], A	-60 men and women, 42 ± 12 years -double-blind, placebo-controlled, parallel design -group 1: CaP (aCa: 1000 mg, bCa: 1014 mg, aP: 500 mg, bP: 1333 mg) -group 2: vitamin D (10 µg/day, bCa: 916 mg, bP: 1324 mg) -group 3: CaP + vitamin D (10 µg, aCa: 1000 mg, bCa: 872 mg, aP: 500 mg, bP: 1205 mg) -8 weeks each intervention -before the interventions, 2 weeks of placebo in each group (bCa: approximately 900 mg, bP: 1300 mg) -supplement: CaP and vitamin D_3_	-uCa ↑ after CaP as compared with placebo -fCa and fP ↑ after CaP and CaP + vitamin D as compared with placebo -uP and pPO_4_ = after CaP as compared with placebo -pCa ↑ after all interventions as compared with placebo	-25-(OH)D ↑ after vitamin D and CaP + vitamin D after 8 weeks→ after 4 weeks only in the CaP + vitamin D group -PTH = after all interventions as compared with placebo -bone markers = after all interventions as compared with placebo
[27] C	*Study 1* -15 men and women, 20.4 ± 1.6 years -double-blind, cross over design -test diet 1: tricalcium phosphate (aCa: 500 mg, aP: 258 mg *) -test diet 2: calcium carbonate (aCa: 500 mg) -2 weeks each diet *Study 2* -17 men and women, 25.2 ± 6.8 years -double-blind, cross over design -test diet 1: 2 probiotic strains -test diet 2: 2 probiotic strains + calcium carbonate (aCa: 500 mg) -test diet 3: 2 probiotic strains + tricalcium phosphate (aCa: 500 mg, aP: 258 mg *) -2 weeks each diet	-Study 1: faecal *Lactobacillus* ssp. counts = -Study 2: faecal *Lactobacillus* ssp. counts ↑ after probiotic + tricalcium phosphate as compared with baseline	Study 2: faecal *Lactobacillus* ssp. counts = after probiotic with calcium carbonate
[28] C	-14 men and women, 25–37 years -single-arm-study -control period 1: habitual diet (bCa: 1400 mg. bP: 1700 mg) -test diet: tricalcium phosphate (Ca_3_(PO_4_)_2_, aCa: 1500 mg, bCa: 1400 mg; aP: 750 mg *, bP: 1700 mg) -control period 2: habitual diet (bCa: 1400 mg, bP: 1700 mg) -1 weeks each period and diet	-cytolytic activity and intestinal alkaline phosphatase of faecal water, fBA, ffats = after tricalcium phosphate	-modulation of duodenal bile acids (no significant effect) after tricalcium phosphate
	**Phosphate supplementation**		
[29] A	-6 men, 26–40 years -single-arm study -control period: normal diet (aCa: 650 mg; bCa: 200 mg; aP: 1000 mg; bP: 500 mg) neutral sodium phosphate/potassium phosphate, calcium carbonate -test period 1: low phosphorus (aCa: 650 mg, bCa: 200 mg, bP: < 500 by phosphate binders), sodium chloride/potassium chloride, calcium carbonate -test period 2: high phosphorus (aCa: 650 mg, bCa: 200 mg, aP: 2500 mg, bP: 500 mg), sodium phosphate/potassium phosphate, calcium carbonate -9 days (control period), 10 days (test periods)	-sPO_4_ ↑ after high phosphorus diet increased after 1 day → ↓within 10 days as compared with control period -uP ↑; uCa ↓; sCa= after high phosphorus diet as compared with control period	-1,25-(OH)_2_D ↑ after low phosphorus diet as compared with control period -1,25-(OH)_2_D ↓ after high phosphorus diet as compared with control period -production rate of 1,25OHD ↑ after low phosphorus diet as compared with control period -production rate of 1,25OHD ↓ after high phosphorus diet as compared with control period -metabolic clearance rate of 1,25OHD=
[30] A	-6 men, 26–40 year -single-arm study -control period: normal diet (aCa: 650 mg; bCa: 200 mg; aP: 1000 mg; bP: 500 mg) neutral sodium phosphate/potassium phosphate, calcium carbonate -test period 1: low phosphorus (aCa: 650 mg, bCa: 200 mg, bP: < 500 mg by phosphate binders), sodium chloride/potassium chloride, calcium carbonate -test period 2: high phosphorus (aCa: 650 mg, bCa: 200 mg, aP: 2500 mg, bP: 500 mg), sodium phosphate/potassium phosphate, calcium carbonate -9 days (control period), 10 days (test periods)	-24 h mean of sPO_4_ ↑, with doubled peak in the afternoon rise after high phosphorus diet as compared with control diet -24 h mean of sCa=, 24 h mean of ionized Ca ↓ after high phosphorus diet as compared with control period	-24 h mean sPO_4_ ↓ after low phosphorus diet as compared with control period
[31] A	-15 young women, 18–25 years -parallel design, -control group: basal diet (bCa: 800 mg, bP: 900 mg), 56 days -experimental group: first 28 days of basal diet (bCa: 800 mg, bP: 900 mg), 28 days of high phosphorus/low calcium diet (bCa: 400 mg, bP: 1700 mg) -supplement: processed food	-sPO_4_ (fasting and postprandial) = -fasting sCa ↓ after high phosphorus/low calcium diet as compared with basal diet -postprandial sCa=	-PTH ↑ after high phosphorus/low calcium diet as compared with basal diet -1,25-(OH)_2_D=
[32] A	-79 postmenopausal women, 50–75 year -double-blind, placebo-controlled, parallel design -group I (aP: 750 mg) -group II (aP: 1500 mg) -group III (aP: 2250 mg) -group IV placebo -supplement: mix of ammonium phosphate, potassium phosphate, and glycerol phosphate -7 days each group	-sPO_4_= -uPO_4_/Crea ↑ with increasing phosphate → returned to normal after 7 days follow-up -uCa/Crea and sCa=	-PTH ↑ in group II/III -1,25-(OH)_2_D= -group II: osteocalcin ↑
[33] A	*Study 1* -10 healthy men, 19–32 years -randomised, cross-over design -control diet (bCa: 500 mg, bPO_4_: 800 mg -test diet (bCa: 500 mg, aP: 1000 mg, bPO_4_: 800 mg) -7 days each diet -supplement: sodium acid phosphate *Study 2* -12 healthy men, 19–38 years -single-arm study -test diet 1: control diet (bCa: 1000 mg, bP: 1000 mg) -test diet 2 (bCa: 1000 mg, aP: 1000 mg, bP: 1000 mg) -test diet 3 (bCa: 1000 mg, aP: 1500 mg, bP: 1000 mg) -test diet 4: (bCa: 1000 mg, aP: 2000 mg, bP: 1000 mg) -7 days each diet -supplement: sodium acid phosphate	-both studies: uPO_4_ ↑ and uCa↓ after phosphorus diets as compared with control diets -sPO_4_ and sCa=	-PTH: Study 1 ↑ after test diet as compared with control diet and Study 2= -no change in bone markers
[34] A	-13 healthy men, 28–43 years -single-arm study -test diet 1: control (bCa: 850 mg, aP: 1000 mg, bP: 500 mg) -test diet 2: supplemented (bCa: 850 mg, aP: 1800 mg, bP: 500 mg) -test diet 3: restricted (bCa: 850 mg, bP: 625 mg) -9 days for each diet -supplement: sodium and potassium phosphate	-uP ↑, uCa=, sPO_4_, sCa = after supplemented diet as compared with control diet	-FGF23 ↓ after restriction diet as compared with supplemented and control diets -1,25-(OH)_2_D ↑ after restriction diet as compared with supplemented and control diets -PTH ↓ after restriction diet as compared with supplemented and control diets
[35] A	-12 healthy men and women (from control group), 31–48 years -cross-over design -test diet 1: high phosphate (bP: 2000 mg) -test diet 2: low phosphate (bP: 750 mg) -test diet 3: low phosphate with phosphate binder (bP: 750 mg + phosphate binder) -7 days each test diet -supplement: non-perishable manufactured food products	-sPO_4_=, uP ↑, uCa = after high phosphate diet as compared with baseline	-FGF23 = after high phosphate diet as compared with baseline -PTH = after high phosphate diet as compared with baseline
[36] A	-10 healthy men and women, 19–45 years -single-arm design -run in period: ad libitum diets -test diet 1: low additive diet (bCa: 732 mg, bP: 1070 mg) -test diet 2: high additive diet (bCa: 677 mg, bP: 1677 mg) -2 weeks (run in period), 1 week (test diets) -supplement: phosphorus additive enhanced menus	-uP↓, uCa=, sPO_4_=, sCa= after high additive diet as compared with run in period	PTH=, FGF23↑, osteocalcin ↑, P1NP ↓, sclerostin ↓, osteopontin ↑ after high additive diet compared low additive diet
[20] A	-62 men and women, 29 ± 7 years -double-blind, placebo-controlled, parallel design -group 1 (bCa: 900 mg, aP: 1000 mg, bP: 1200 mg) -group 2: (aCa: 500 mg, bCa: 900 mg, aP: 1000 mg, bP: 1300 mg) -group 3 (aCa: 1000 mg, bCa: 900 mg, aP: 1000 mg, bP: 1300 mg) -8 weeks each intervention -before the interventions, 2 weeks of placebo in each group (bCa: appr. 900 mg, bP: 1300 mg) -supplement: calcium carbonate and monosodium phosphate	-sPO_4_ and sCa= -fP ↑, uP ↑, uCa↓, fCa = after intervention in group 1 (phosphate without calcium) as compared with placebo -fP↑, fCa↑, uCa = in group 2 and 3 (phosphate with calcium)	-FGF23 ↑ after all interventions as compared with placebo after four weeks and then returned to placebo values -bone marker= -PTH=
[13] C	-62 men and women, 29 ± 7 years -double-blind, placebo-controlled, parallel design -group 1 (bCa: 900 mg, aP: 1000 mg, bP: 1200 mg) -group 2: (aCa: 500 mg, bCa: 900 mg, aP: 1000 mg, bP: 1300 mg) -group 3 (aCa: 1000 mg, bCa: 900 mg, aP: 1000 mg, bP: 1300 mg) -8 weeks each intervention -before the interventions, 2 weeks of placebo in each group (bCa: appr. 900 mg, bP: 1300 mg) -supplement: calcium carbonate and monosodium phosphate	-geno- and cytotoxicity of FW = in all groups	-modulation in faecal short-chain fatty acids and microbiota
[37] A	-10 young women, 20–30 years -single-arm design -control period 1: basic diet (bCa: 1500 mg, bP: 1700 mg) -supplementation period: (bCa: 1995 mg, aP: 1600 mg, bP: 1400 mg) -control period 2: basic diet (bCa: 1500 mg, bP: 1700 mg) -2 weeks (control period 1), 6 weeks (supplementation period), 4 weeks (control period 2) -supplement: monosodium phosphate	-sPO_4_=	PTH, bone marker=

[]: number of reference in the text; A: allocation to mineral metabolism; B: allocation to blood lipids; C: allocation to intestinal parameters; ^1^ presented results are only those in relation to the aim of the present investigation; ^2^ further results/comments according to the aim of the respective study; * estimated phosphorus intake; s, s: serum, p: plasma, a: additional, b: baseline, u: urine, f: faecal, =: no change, ↑: significant increase, ↓: significant decrease, AUC: area under the curve, P: phosphorus, PO4: phosphate, Ca: calcium, TCP: ossein-hydroxyapatit, OHC: tricalcium phosphate, PTH: parathyroid hormone, 1,25-(OH)_2_D: 1,25-dihydroxy-cholecalciferol, 25-(OH)D: 25-hydroxycholecalciferol, BA: bile acids:, Crea: creatinine, NS: neutral sterol; FGF23: fibroblast growth factor 23, CFU: colony forming units, CaP: pentacalcium hydroxy-trisphosphate, LCA: lithocholic acid, iLCA: iso lithocholic acid, DCA: deoxycholic acid, 12keto DCA: 12keto deoxycholic acid.

## Supplementary Materials

The following are available online at http://www.mdpi.com/2072-6643/10/7/936/s1, Figure S1: Effect of CaP supplementation on urine and faecal calcium and phosphorus excretion in men, Figure S2: Effect of CaP supplementation on faecal excretion of bile acid and neutral sterols in men, Figure S3: Effect of CaP supplementation on urine and faecal calcium and phosphorus excretion in women, Figure S4: Effect of CaP supplementation on faecal excretion of bile acid and neutral sterols in women, Table S1: Dietary intake of macronutrients as well as calcium and phosphorus in men and women after CaP supplementation, Table S2: Effect of CaP supplementation on blood lipids in men and women, Table S3: Effect of CaP supplementation on faecal bile acids in men and women, Table S4. Effect of CaP supplementation on the faecal excretion of neutral sterols in men and women.
