# Supplementary material for: Calcium and Phosphate Metabolism, Blood Lipids and Intestinal Sterols in Human Intervention Studies Using Different Sources of Phosphate as Supplements—Pooled Results and Literature Search
Source: Nutrients. 2018 Jul 20;10(7):936. doi: 10.3390/nu10070936 (PMC6073240; doi:10.3390/nu10070936)
Supplement: Supplementary file 1 [file nutrients-10-00936-s001.docx]

**Supplementary materials:**

**Table S1.** Dietary intake of macronutrients as well as calcium and phosphorus in men and women after CaP supplementation.−

| **Gender** | **Placebo** | | | **CaP** | | | **Change** | | |
| --- | --- | --- | --- | --- | --- | --- | --- | --- | --- |
| **Energy [MJ/d]** | | | | | | | | | |
| Men | 10 ± 1 | | | 10 ± 2 | | | 0.1 ± 1 | | |
|  | (10–11) | | | (10–11) | | | (−0.2–0.4) | | |
| Women | 8 ± 1 | | | 8 ± 1 | | | 0.1 ± 0.9 | | |
|  | (7–8) | | | (7–8) | | | (−0.2–0.4) | | |
| **Fat [g/d]** | | | | | | | | | |
| Men | 101 | ± | 20 | 102 | ± | 22 | 0.4 | ± | 18 |
|  | (95–107) | | | (95–108) | | | (−5–6) | | |
| Women | 65 | ± | 15 | 66 | ± | 17 | 1 | ± | 11 |
|  | (60–70) | | | (61–71) | | | (−2–4) | | |
| **Protein [g/d]** | | | | | | | | | |
| Men | 95 | ± | 15 | 94 | ± | 15 | −1 | ± | 14 |
|  | (91–99) | | | (90–98) | | | (−5–3) | | |
| Women | 67 | ± | 12 | 69 | ± | 13 | 1 | ± | 10 |
|  | (64–71) | | | (65–73) | | | (−2–5) | | |
| **Carbohydrates [g/d]** | | | | | | | | | |
| Men | 288 | ± | 46 | 294 | ± | 51 | 6 | ± | 37 |
|  | (274–301) | | | (279–309) | | | (−4–17) | | |
| Women | 240 | ± | 40 | 244 | ± | 49 | 4 | ± | 36 |
|  | (228–252) | | | (228–258) | | | (−8–15) | | |
| **Calcium [mg/d]** | | | | | | | | | |
| Men | 1176 | ± | 302 | 2204* | ± | 343 | 1029^#^ | ± | 235 |
|  | (1088–1263) | | | (2104–2304) | | | (960–1097) | | |
| Women | 948 | ± | 220 | 1956* | ± | 214 | 1008^#^ | ± | 177 |
|  | (879–1016) | | | (1889–2022) | | | (953–1063) | | |
| **Calcium [mg/kg bw]** | | | | | | | | | |
| Men | 16 | ± | 4 | 30* | ± | 5 | 14^#^ | ± | 4 |
|  | (15–17) | | | (28–31) | | | (13–15) | | |
| Women | 16 | ± | 4 | 32* | ± | 5 | 17^#^ | ± | 4 |
|  | (14–17) | | | (31–34) | | | (16–18) | | |
| **Phosphorus [mg/d]** | | | | | | | | | |
| Men | 1628 | ± | 261 | 2104* | ± | 307 | 476^#^ | ± | 234 |
|  | (1552–1703) | | | (2015–2193) | | | (408–544) | | |
| Women | 1213 | ± | 207 | 1719* | ± | 196 | 506^#^ | ± | 149 |
|  | (1148–1277) | | | (1658–1780) | | | (460–553) | | |
| **Phosphorus [mg/kg bw]** | | | | | | | | | |
| Men | 22 | ± | 5 | 28* | ± | 5 | 6^#^ | ± | 3 |
|  | (21–23) | | | (27–30) | | | (5–7) | | |
| Women | 20 | ± | 4 | 28* | ± | 5 | 8^#^ | ± | 3 |
|  | (19–21) | | | (27–30) | | | (8–9) | | |

Men: *n* (studies) = 4, *n* (subjects) = 48; women: *n* (studies) = 3, *n* (subjects) = 42; mean ± standard deviation; (95% confidence interval); CaP: three - four weeks´ intervention with Ca_5_(PO_4_)_3_OH (pentacalcium hydroxy-trisphosphate); bw, body weight; * significantly different to placebo (paired Student’s t-test, *p* ≤ 0.05); # significantly different to zero (one-way ANOVA, *p* ≤ 0.05).

**Table S2.** Effect of CaP supplementation on blood lipids in men and women.

| **Gender** | **Placebo** | | | **CaP** | | | **Change** | | |
| --- | --- | --- | --- | --- | --- | --- | --- | --- | --- |
| **Total cholesterol [mmol/l]** | | | | | | | | | |
| Men | 4.54 | ± | 0.95 | 4.43 | ± | 1.01 | −0.12 | ± | 0.43 |
|  | (4.2–4.9) | | | (4.1–4.8) | | | (−0.3–0.02) | | |
| Women | 5.03 | ± | 1.12 | 4.86 | ± | 1.02 | −0.17 | ± | 0.51 |
|  | (4.7–5.4) | | | (4.5–5.2) | | | (−3.3–(−0.02)) | | |
| **HDL−cholesterol [mmol/l]** | | | | | | | | | |
| Men | 1.24 | ± | 0.27 | 1.28 | ± | 0.27 | 0.03 | ± | 0.20 |
|  | (1.2–1.3) | | | (1.2–1.4) | | | (−0.03–0.1) | | |
| Women | 1.65 | ± | 0.35 | 1.67 | ± | 0.37 | 0.02 | ± | 0.14 |
|  | (1.5–1.8) | | | (1.6–1.8) | | | (−0.02–0.7) | | |
| **LDL−cholesterol [mmol/l]** | | | | | | | | | |
| Men | 2.87 | ± | 0.82 | 2.77* | ± | 0.87 | −0.10 | ± | 0.36 |
|  | (2.6–3.1) | | | (2.5–3.0) | | | (−0.2–0.01) | | |
| Women | 2.87 | ± | 1.06 | 2.75 | ± | 0.98 | −0.12^#^ | ± | 0.47 |
|  | (2.5–3.2) | | | (3.4–3.1) | | | (−0.27–0.03) | | |
| **LDL:HDL−ratio** | | | | | | | | | |
| Men | 2.41 | ± | 0.92 | 2.25 | ± | 0.83 | −0.16 | ± | 0.50 |
|  | (2.1–2.7) | | | (2.0–2.5) | | | (−0.33–0) | | |
| Women | 1.85 | ± | 0.82 | 1.76 | ± | 0.78 | −0.09^#^ | ± | 0.31 |
|  | (1.6–2.1) | | | (1.5–1.2) | | | (−0.2–0) | | |
| **Triacylglycerides [mmol/l]** | | | | | | | | | |
| Men | 0.95 | ± | 0.55 | 0.94 | ± | 0.42 | 0 | ± | 0.53 |
|  | (0.8–1.1) | | | (0.8–1.1) | | | (−0.18–0.17) | | |
| Women | 1.04 | ± | 0.63 | 0.98 | ± | 0.31 | −0.06 | ± | 0.55 |
|  | (0.8–1.2) | | | (0.9–1.1) | | | (−0.2–0.1) | | |

*n* (studies) = 3; men: *n* = 39; women: *n* = 42; mean ± standard deviation; (95% confidence interval); CaP: three - four weeks´ intervention with Ca_5_(PO_4_)_3_OH (pentacalcium hydroxy-trisphosphate); * significantly different to placebo (paired Student’s t-test, *p* ≤ 0.05); # significantly different to zero (one-way ANOVA, *p* ≤ 0.05).

**Table S3.** Effect of CaP supplementation on faecal bile acids in men and women.

| **Gender** | **Placebo** | | | **CaP** | | | **Absolute change** | | |
| --- | --- | --- | --- | --- | --- | --- | --- | --- | --- |
| **iLCA [mg/d]** | | | | | | | | | |
| Men | 46 | ± | 27 | 52 | ± | 32 | 6 | ± | 23 |
|  | (36–56) | | | (40–63) | | | (−3–14) | | |
| Women | 32 | ± | 19 | 38 | ± | 23 | 6 | ± | 19 |
|  | (25–39) | | | (29–46) | | | (−1.3–12.6) | | |
| **LCA [mg/d]** | | | | | | | | | |
| Men | 66 | ± | 37 | 83* | ± | 43 | 17 | ± | 31 |
|  | (52–79) | | | (67–98) | | | (6–28) | | |
| Women | 43 | ± | 17 | 55* | ± | 21 | 12^#^ | ± | 15 |
|  | (37–49) | | | (47–63) | | | (6–18) | | |
| **iDCA [mg/d]** | | | | | | | | | |
| Men | 34 | ± | 17 | 37 | ± | 23 | 3 | ± | 18 |
|  | (28–40) | | | (28–45) | | | (−4–9) | | |
| Women | 34 | ± | 30 | 37 | ± | 52 | 3 | ± | 35 |
|  | (22–45) | | | (18–56) | | | (−10–16) | | |
| **DCA [mg/d]** | | | | | | | | | |
| Men | 109 | ± | 46 | 154* | ± | 91 | 45 | ± | 71 |
|  | (92–126) | | | (121–188) | | | (19–71) | | |
| Women | 88 | ± | 36 | 115* | ± | 41 | 28^#^ | ± | 33 |
|  | (74–101) | | | (100–130) | | | (15–40) | | |
| **CDCA [mg/d]** | | | | | | | | | |
| Men | 7 | ± | 4 | 8 | ± | 5 | 1^#^ | ± | 5 |
|  | (5–8) | | | (6–1) | | | (−0.8–3) | | |
| Women | 5 | ± | 4 | 6 | ± | 5 | 1 | ± | 4 |
|  | (4–6) | | | (5–8) | | | (−0.1–2.7) | | |
| **CA [mg/d]** | | | | | | | | | |
| Men | 9 | ± | 7 | 13 | ± | 13 | 3 | ± | 12 |
|  | (7–12) | | | (8–18) | | | (−1–8) | | |
| Women | 11 | ± | 16 | 12 | ± | 9 | 0.5 | ± | 10 |
|  | (5–17) | | | (8–15) | | | (−3–4) | | |
| **12keto DCA [mg/d]** | | | | | | | | | |
| Men | 10 | ± | 8 | 16* | ± | 16 | 6 | ± | 12 |
|  | (7–13) | | | (10–22) | | | (2–10) | | |
| Women | 6 | ± | 3 | 7* | ± | 4 | 2 | ± | 4 |
|  | (4–7) | | | (6–9) | | | (0–3) | | |

*n* (studies) = 2, men: *n* = 31; women: *n* = 31; mean ± standard deviation; (95% confidence interval); CaP: three - four weeks´ intervention with Ca_5_(PO_4_)_3_OH (pentacalcium hydroxy-trisphosphate); * significantly different to placebo (paired Student’s t-test, *p* ≤ 0.05); # significantly different to zero (one-way ANOVA, *p* ≤ 0.05).

**Table S4.** Effect of CaP supplementation on the faecal excretion of neutral sterols in men and women.

| **Gender** | **Placebo** | | | **CaP** | | | **Change** | | |
| --- | --- | --- | --- | --- | --- | --- | --- | --- | --- |
| **Coprostanol [mg/d]** | | | | | | | | | |
| Men | 406 | ± | 236 | 459 | ± | 274 | 53 | ± | 250 |
|  | (320–493) | | | (358–559) | | | (−39–144) | | |
| Women | 452 | ± | 290 | 429 | ± | 249 | −23 | ± | 227 |
|  | (352–552) | | | (343–514) | | | (−101–54) | | |
| **Cholesterol [mg/d]** | | | | | | | | | |
| Men | 169 | ± | 150 | 195 | ± | 223 | 26 | ± | 114 |
|  | (114–223) | | | (113–277) | | | (−15–68) | | |
| Women | 152 | ± | 157 | 140 | ± | 146 | −12 | ± | 126 |
|  | (99–206) | | | (90–190) | | | (−56–31) | | |
| **Cholestanol [mg/d]** | | | | | | | | | |
| Men | 13 | ± | 8 | 13 | ± | 9 | −0.4 | ± | 6 |
|  | (11–16) | | | (10–16) | | | (2–2) | | |
| Women | 14 | ± | 8 | 14 | ± | 6 | −1 | ± | 4 |
|  | (12–17) | | | (11–17) | | | (−2–1) | | |
| **Coprostanone [mg/d]** | | | | | | | | | |
| Men | 40 | ± | 36 | 38 | ± | 26 | −2 | ± | 30 |
|  | (27 − 53) | | | (28–48) | | | (−13–9) | | |
| Women | 42 | ± | 49 | 42 | ± | 39 | 0 | ± | 37 |
|  | (25–58) | | | (28–55) | | | (−13–13) | | |
| **Cholestanone [mg/d]** | | | | | | | | | |
| Men | 3 | ± | 2 | 4 | ± | 2 | 1 | ± | 2 |
|  | (2–4) | | | (3–4) | | | (0–1) | | |
| Women | 3 | ± | 3 | 3 | ± | 2 | 0 | ± | 2 |
|  | (2 − 4) | | | (2–3) | | | (−1–0) | | |
| **Cholestenone [mg/d]** | | | | | | | | | |
| Men | 5 | ± | 4 | 6 | ± | 5 | 1 | ± | 3 |
|  | (4–7) | | | (4–8) | | | (−1–2) | | |
| Women | 5 | ± | 4 | 5 | ± | 3 | 0 | ± | 4 |
|  | (4–7) | | | (4–6) | | | (−2–1) | | |

*n* (studies) = 3; men: *n* = 31; women: *n* = 35; mean ± standard deviation; (95% confidence interval); * significantly different to placebo (paired Student’s t-test, *p* ≤ 0.05); # significantly different to zero (one-way ANOVA, *p* ≤ 0.05); CaP: three - four weeks´ intervention with Ca_5_(PO_4_)_3_OH (pentacalcium hydroxy-trisphosphate).


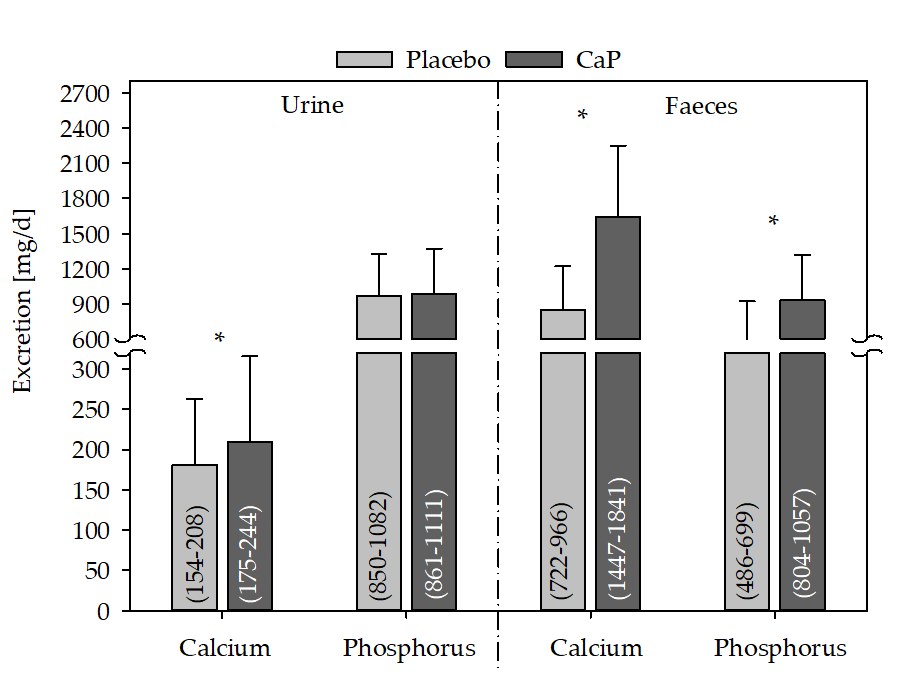


**Figure S1.** Effect of CaP supplementation on urine and faecal calcium and phosphorus excretion in men. *n* (studies) = 3; *n* (subjects) = 39; mean + standard deviation; (95% confidence interval); * significantly different to placebo (paired Student’s t-test, *p* ≤ 0.05); CaP: three - four weeks´ intervention with Ca_5_(PO_4_)_3_OH (pentacalcium hydroxy-trisphosphate).


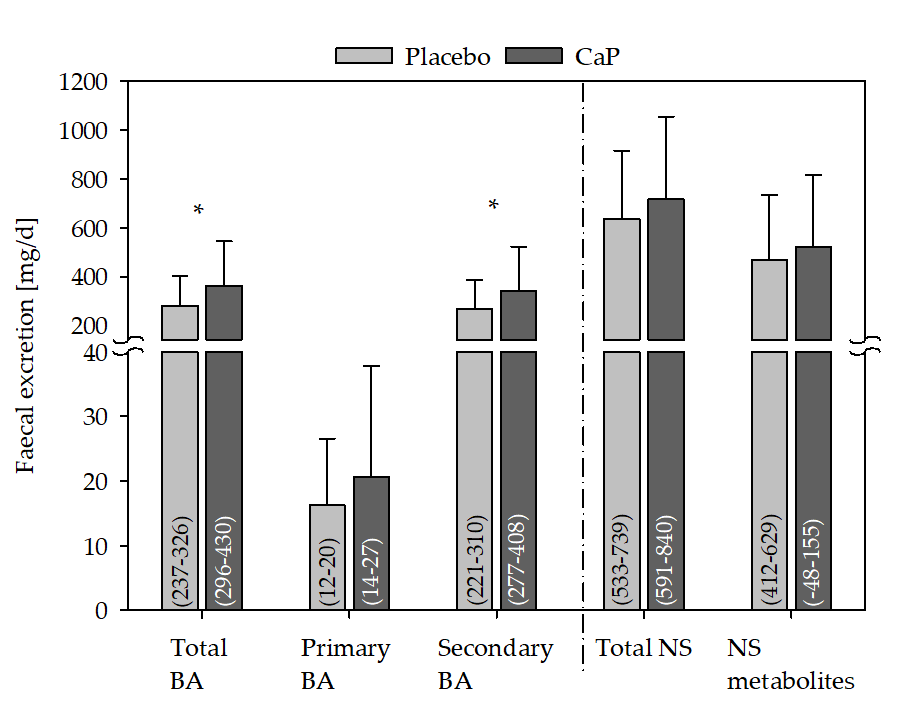


**Figure S2.** Effect of CaP supplementation on faecal excretion of bile acid and neutral sterols in men. BA: n (studies) = 2, n (subjects) = 31; NS: n (studies) = 3, n (subjects) = 31; mean + standard deviation; (95% confidence interval); * significantly different to placebo (paired Student’s t-test, p ≤ 0.05); CaP: three - four weeks´ intervention with Ca_5_(PO_4_)_3_OH (pentacalcium hydroxy-trisphosphate); BA: bile acids; NS: neutral sterols.


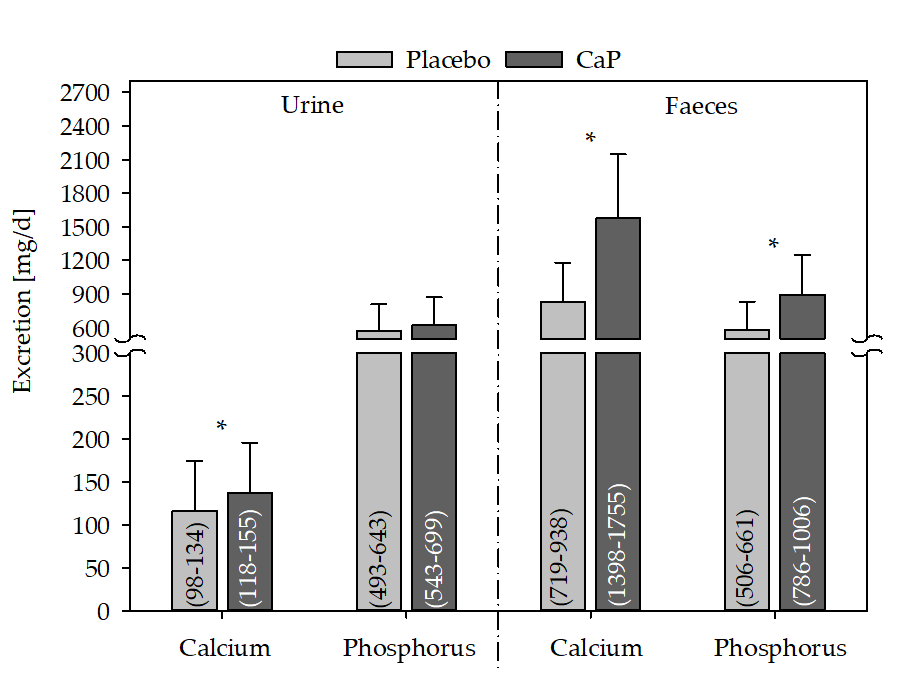


**Figure S3.** Effect of CaP supplementation on urine and faecal calcium and phosphorus excretion in women. *n* (studies) = 3; *n* (subjects) = 42; mean + standard deviation; (95% confidence interval); * significantly different to placebo (paired Student’s t-test, *p* ≤ 0.05); CaP: three–four weeks´ intervention with Ca_5_(PO_4_)_3_OH (pentacalcium hydroxy-trisphosphate).

**
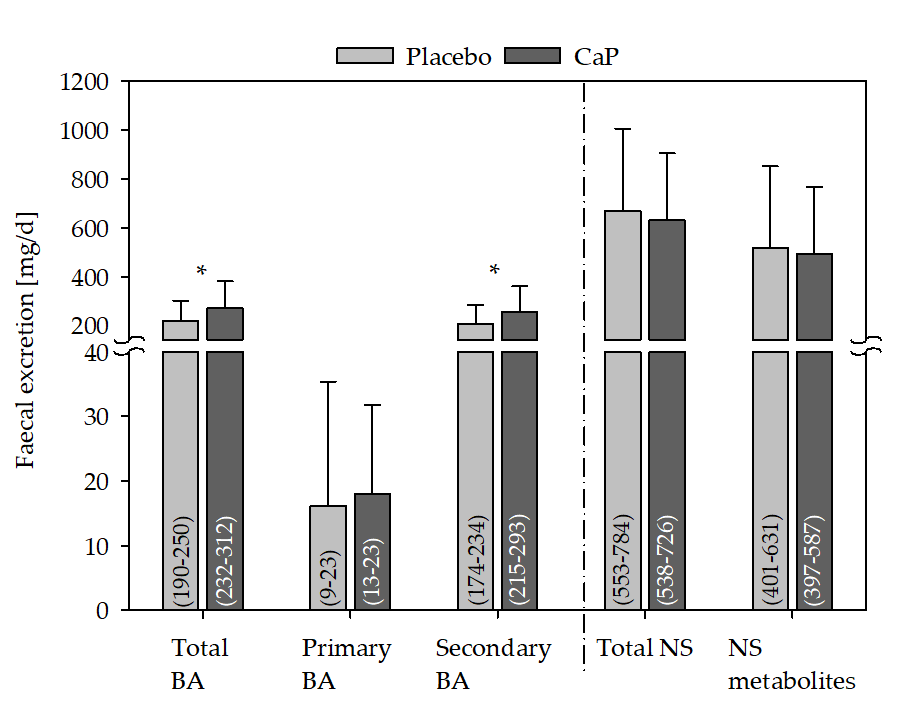
**

**Figure S4.** Effect of CaP supplementation on faecal excretion of bile acid and neutral sterols in women. BA: *n* (studies) = 2, *n* (subjects) = 31; NS: *n* (studies) = 3, *n* (subjects) = 35 (neutral sterols); mean + standard deviation; (95% confidence interval); * significantly different to placebo (paired Student’s t-test, *p* ≤ 0.05); CaP: three - four weeks´ intervention with Ca_5_(PO_4_)_3_OH (pentacalcium hydroxy-trisphosphate); BA: bile acids; NS: neutral sterols.
